# Supplementary material for: Plant-soil feedbacks help explain biodiversity-productivity relationships
Source: Commun Biol. 2021 Jun 25;4:789. doi: 10.1038/s42003-021-02329-1 (PMC8233354; doi:10.1038/s42003-021-02329-1)
Supplement: Supplementary file 2 — Supplemental materials [file 42003_2021_2329_MOESM2_ESM.pdf]

Supplementary Information

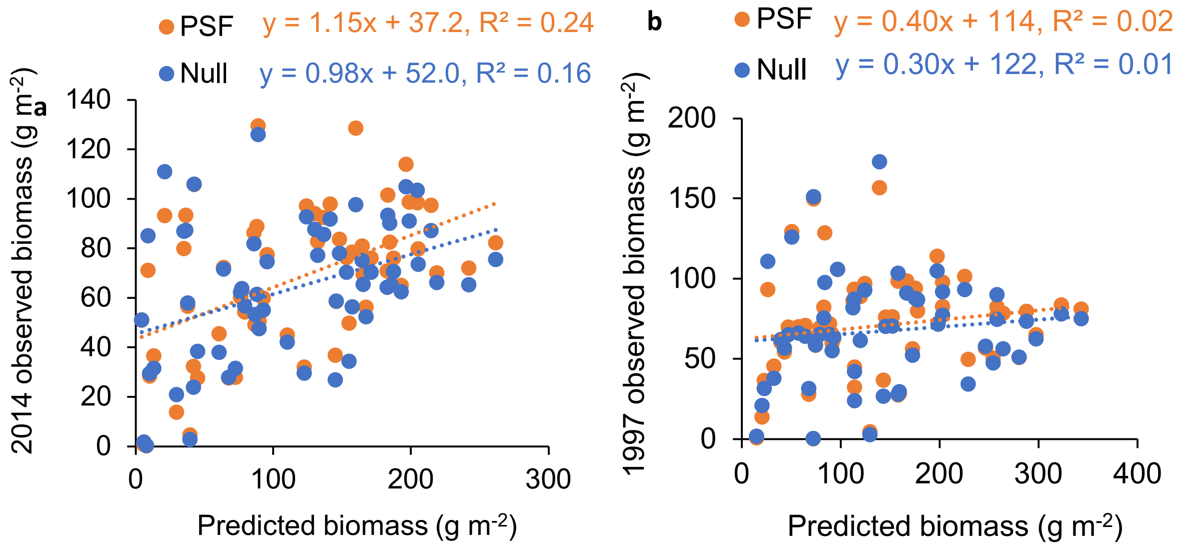

Supplementary Figure 1. Observed versus predicted biomass of communities with one to 16 species. Experimental plant communities with one to 16 plant species were planted in 2014 (a; n = 60) and 1997 (b; n = 56) and maintained for four years. Biomass in these communities (observed) was compared to predictions from plant community simulation models that either included plant-soil feedbacks (PSF) or not (Null). Best fit lines are shown, but were only significant for the 2014 dataset.

Supplementary Table 1. Treatments and sample sizes for the plant-soil feedback experiment. Each Phase I conditioned soil type had between five and nine 'control' plots, between 27 and 30 'self' plots, and between five and nine 'other' plots per Phase II species for a total of between 75 and 135 'other' plots. 112 plots that did not have seeded species growth in Phase I, i.e. Phase I control treatment, were seeded with either one of the sixteen target species (N = 96) or left unseeded (N = 16).

Phase I	Phase II	N
Ac	Control	5
Ac	Self	34
Ac	Other	120
Ag	Control	5
Ag	Self	30
Ag	Other	135
Am	Control	5
Am	Self	30
Am	Other	135
Control	Self	16
Control	Other	96
Dp	Control	5
Dp	Self	30
Dp	Other	135
Ec	Control	5
Ec	Self	30
Ec	Other	135
Km	Control	5
Km	Self	30
Km	Other	135
La	Control	5
La	Self	34
La	Other	120
Lc	Control	5
Lc	Self	30
Lc	Other	135
Lp	Control	5
Lp	Self	30
Lp	Other	120
Mf	Control	5
Mf	Self	34
Mf	Other	120
Pp	Control	5
Pp	Self	28
Pp	Other	135

Phase I	Phase II	N
Ps	Control	5
Ps	Self	31
Ps	Other	135
Pv	Control	5
Pv	Self	30
Pv	Other	135
Sn	Control	5
Sn	Self	30
Sn	Other	135
Sr	Control	5
Sr	Self	27
Sr	Other	75
Ss	Control	5
Ss	Self	30
Ss	Other	135

Supplementary Note 1. Modeling details for plant species biomass in communities.

Plant species biomass in communities was predicted using logistic growth simulation models[1] (Supplementary Table 1). Species growth rates were derived from a) growth on control soils (control Null model), b) growth on 'self' soils (self Null model), or c) growth on all soil types (PSF model). Competition coefficients were assigned a value of '1', but each species could affect the growth of other species due to community-level carrying capacities. Each of these three models was run with five different carrying capacities: 1) the maximum observed growth in any plot in the community experiment, 2) the maximum mean observed growth in any community, 3) the maximum species-specific growth in community plots, 4) the maximum observed growth in any PSF plot, and 5) the maximum species-specific growth in any PSF plot. Mean Null model predictions of community biomass were calculated from the 10 model simulations (Control Null, Self Null each with five carrying capacities. Mean PSF model predictions were calculated from the five simulations with different carrying capacities.

Plant growth rates were calculated from the initial seed mass (0.002 g) and final observed biomass of each species on each soil. For example, for a model with 52 time steps, the growth rate of species A on soil α is

$$\Gamma_{A\alpha} = \sqrt[52]{A\alpha/I} \tag{1}$$

where Aα = the final biomass of plant A on soil α, and I = initial seed mass (Supplementary Table 3). A different growth rate is calculated for each plant species on each species-conditioned soil (i.e., soil type):

$$\Gamma_{A_t} = \Gamma_{A\alpha}P\alpha + \Gamma_{A\beta}P\beta + \cdots + \Gamma_{A\iota}P\iota \tag{2}$$

$$\Gamma_{B_t} = \Gamma_{B\alpha}\,P\alpha + \Gamma_{B\beta}\,P\beta + \cdots + \Gamma_{B\iota}\,P\iota \tag{2a}$$

$$\vdots$$

$$\Gamma_{I_t} = \Gamma_{I\alpha}P\alpha + \Gamma_{I\beta}P\beta + \cdots + \Gamma_{I\iota}P\iota \tag{2n}$$

Species-conditioned soils 'grow' as a function of plant biomass, plant species growth rates, and a conversion factor μ (Table S1). Conversion factor μ was set to 5 to reflect the assumption that microbial communities grow faster than plants[1]. Species-conditioned soil growth was modeled as

$$\alpha_{t+1} = \left(1 + \mu\Gamma_{A_t}A_t\right)\alpha_t,\ \beta_{t+1} = \left(1 + \mu\Gamma_{B_t}B_t\right)\beta_t\ , \ldots, \iota_{t+1} = \left(1 + \mu\Gamma_{I_t}I_t\right)\iota_t. \tag{3}$$

The proportion each conditioned soil type comprises of the total soil community can be described by

$$P_{\alpha_t} = \alpha_t/(\alpha_t + \beta_t + \cdots + \iota_t) \tag{4}$$

(Table S1). Plant growth rates are a function of the proportion of different conditioned soil types present. To prevent run-away growth, biomass is limited by a carrying capacity κ, which can be either unique to a species or to the community (Table S1). Changes in each plant's biomass can be described as

$$A_{t+1} = A_t + \Gamma_{A_t}\left((\kappa - A_t)/\kappa\right) \quad (5a)$$

$$B_{t+1} = B_t + \Gamma_{B_t}\left((\kappa - B_t)/\kappa\right) \quad (5b)$$

$$\ldots, I_{t+1} = I_t + \Gamma_{I_t}\left((\kappa - I_t)/\kappa\right) \quad (5c)$$

Although the Null models are similar in their implementation, they do not incorporate growth on all conditioned soil types. For the self Null model, plant species biomass is a function of observed plant biomass on "self" soil only, i.e.

$$A = f(\Gamma_{A\alpha}) \quad (6a)$$

$$B = f(\Gamma_{B\beta}) \quad (6b)$$

$$\ldots$$

$$I = f(\Gamma_{I\iota}) \quad (6n)$$

(Supplementary Table 2}). For the control Null model, plant species biomass is a function of observed plant biomass on unconditioned control soils only, i.e.

$$A = f(\Gamma_{A_{cntl}}) \quad (7a)$$

$$B = f(\Gamma_{B_{cntl}}) \quad (7a)$$

$$\ldots$$

$$I = f(\Gamma_{I_{cntl}}) \quad (7a)$$

(Supplementary Table 2).

Because growth rates were derived from the second year of growth, we assumed that growth rates represented two years of growth. To simulate the four years of growth in the biodiversity-productivity experiment, model simulations were executed for 52 timesteps, after which plant biomass was reduced to 1% of the previous timestep and allowed to run for another 52 timesteps. Mean model output for the sum of species growth from the suite of Null or PSF model simulations are reported.

Supplementary Table 2. Parameter definitions for the plant community simulation models.

Parameter	Definition
A, B, …, I	Plant A through I
A_t, B_t, …, I_t	Biomass of plant A through I at time t

$\alpha, \beta, \ldots, \iota$	Conditioned soil types α through ι, cultivated by plants A through I
$\Gamma_{At}, \Gamma_{Bt}, \ldots, \Gamma_{It}$	Growth rate of plant A through I at time t
$P_{\alpha}, P_{\beta}, \ldots, P_{\iota}$	Proportion of conditioned soil type α through ι
μ	Conversion factor
κ	Carrying capacity
$A_{cntl}, B_{cntl}, \ldots, I_{cntl}$	Plant A through I's biomass on unconditioned soil
$A\alpha, A\beta, \ldots, A\iota$	Plant A through I's biomass on conditioned soil types α through ι
$B\alpha, B\beta, \ldots, B\iota$	
$\vdots$	
$I\alpha, I\beta, \ldots, I\iota$	
$\Gamma_{A\alpha}, \Gamma_{A\beta}, \ldots, \Gamma_{A\iota}$	Growth rates of species A through I on conditioned soil types α through ι
$\Gamma_{B\alpha}, \Gamma_{B\beta}, \ldots, \Gamma_{B\iota}$	
$\vdots$	
$\Gamma_{I\alpha}, \Gamma_{I\beta}, \ldots, \Gamma_{I\iota}$	

Supplementary References

1. Kulmatiski, A., Heavilin, J. & Beard, K. H. Testing predictions of a three-species plant-soil feedback model. *J. Ecol.* **99**, 542–550 (2011).
