# Supplementary material for: Plant-soil feedbacks help explain biodiversity-productivity relationships
Source: Commun Biol. 2021 Jun 25;4:789. doi: 10.1038/s42003-021-02329-1 (PMC8233354; doi:10.1038/s42003-021-02329-1)
Supplement: Supplementary file 3 — Description of Supplementary Files [file 42003_2021_2329_MOESM3_ESM.pdf]

Description of Additional Supplementary Files

File name: Supplementary Data 1

Description:

The first digit indicates whether the species is planted (1) or not planted (0) in the BP1997 community. The second digit indicate whether a species is planted (1) or not planted (0) in the BP community. Thus, a 1 1 indicates that the species occurs in the mixture in both experiments, a 0 0 indicates that the species occurs in the mixture in neither experiment, a 1 0 indicates it was planted in the mixture in BP1997 but not in BP, and a 0 1 indicates it was planted in BP but not in BP1997. Codes indicate the reason the community was changed from the original community in BP1997. Code A indicates no difference between the BP1997 and BP communities. Code B indicates that Dalea candida and Dalea villosa were removed from the community when seeding BP plots. Code C indicates that woody species were removed from the community when seeding BP plots. Code D indicates Asclepias tuberosa was removed from the community when seeding BP plots. For one community, after woody species and A. tuberosa were removed, the diversity would be 7 species, so S. scoparium was randomly selected to create a diversity of 8 species; this community is indicated by code CDE. Two novel communities, community A and community B were created specifically for the BP study because M. fistulosa and S. rigida co-occur in the intended monoculture plots in Biodiversity II due to a seeding error.
