# Supplementary material for: Plant-soil feedbacks help explain biodiversity-productivity relationships
Source: Commun Biol. 2021 Jun 25;4:789. doi: 10.1038/s42003-021-02329-1 (PMC8233354; doi:10.1038/s42003-021-02329-1)
Supplement: Supplementary file 5 — Reporting Summary [file 42003_2021_2329_MOESM5_ESM.pdf]

nature research

Corresponding author(s): *Double-blind peer review submissions: write DBPR and your manuscript number here instead of author names.*

Last updated by author(s): *YYYY-MM-DD*

Reporting Summary

Nature Research wishes to improve the reproducibility of the work that we publish. This form provides structure for consistency and transparency in reporting. For further information on Nature Research policies, see our Editorial Policies and the Editorial Policy Checklist.

Statistics

For all statistical analyses, confirm that the following items are present in the figure legend, table legend, main text, or Methods section.

n/a	Confirmed	
☐	☒	The exact sample size (n) for each experimental group/condition, given as a discrete number and unit of measurement
☐	☒	A statement on whether measurements were taken from distinct samples or whether the same sample was measured repeatedly
☐	☒	The statistical test(s) used AND whether they are one- or two-sided<br>*Only common tests should be described solely by name; describe more complex techniques in the Methods section.*
☐	☒	A description of all covariates tested
☐	☒	A description of any assumptions or corrections, such as tests of normality and adjustment for multiple comparisons
☐	☒	A full description of the statistical parameters including central tendency (e.g. means) or other basic estimates (e.g. regression coefficient) AND variation (e.g. standard deviation) or associated estimates of uncertainty (e.g. confidence intervals)
☐	☒	For null hypothesis testing, the test statistic (e.g. F, t, r) with confidence intervals, effect sizes, degrees of freedom and P value noted<br>*Give P values as exact values whenever suitable.*
☒	☐	For Bayesian analysis, information on the choice of priors and Markov chain Monte Carlo settings
☒	☐	For hierarchical and complex designs, identification of the appropriate level for tests and full reporting of outcomes
☐	☒	Estimates of effect sizes (e.g. Cohen's d, Pearson's r), indicating how they were calculated

Our web collection on statistics for biologists contains articles on many of the points above.

Software and code

Policy information about availability of computer code

Data collection	Data was collected by hand and entered into Excel spreadsheets.
Data analysis	R (bootstrapped PSF values, ANOVA and post-hoc tukey test), SAS (log regressions), Excel (plant-soil feedback models).

For manuscripts utilizing custom algorithms or software that are central to the research but not yet described in published literature, software must be made available to editors and reviewers. We strongly encourage code deposition in a community repository (e.g. GitHub). See the Nature Research guidelines for submitting code & software for further information.

Data

Policy information about availability of data

All manuscripts must include a data availability statement. This statement should provide the following information, where applicable:

- Accession codes, unique identifiers, or web links for publicly available datasets
- A list of figures that have associated raw data
- A description of any restrictions on data availability

All data are archived with a DOI at Utah State University Digital Commons online repository (https://doi.org/10.26078/52k0-jr94).

Field-specific reporting

Ecological, evolutionary & environmental sciences study design

All studies must disclose on these points even when the disclosure is negative.

Study description	A paired diversity-productivity experiment and two-phase plant-soil feedback experiment

Research sample

Plant-soil feedback experiment
Each Phase I conditioned soil type had between five and nine 'control' plots, between 27 and 30 'self' plots, and between five and nine 'other' plots per Phase II species for a total of between 75 and 135 'other' plots. 112 plots that did not have seeded species growth in Phase I, i.e. Phase I control treatment, were seeded with either one of the sixteen target species (N = 96) or left unseeded (N = 16).
Phase I Phase II N
Amorpha canescens Control 5
Amorpha canescens Self 34
Amorpha canescens Other 120
Andropogon gerardii Control 5
Andropogon gerardii Self 30
Andropogon gerardii Other 135
Achillea millefolium Control 5
Achillea millefolium Self 30
Achillea millefolium Other 135
Unplanted Unconditioned 16
Unplanted Conditioned 96
Dalea purpurea Control 5
Dalea purpurea Self 30
Dalea purpurea Other 135
Elymus canadensis Control 5
Elymus canadensis Self 30
Elymus canadensis Other 135
Koeleria macrantha Control 5
Koeleria macrantha Self 30
Koeleria macrantha Other 135
Liatris aspera Control 5
Liatris aspera Self 34
Liatris aspera Other 120
Lespedeza capitata Control 5
Lespedeza capitata Self 30
Lespedeza capitata Other 135
Lupinus perennis Control 5
Lupinus perennis Self 30
Lupinus perennis Other 120
Monarda fistulosa Control 5
Monarda fistulosa Self 34
Monarda fistulosa Other 120
Poa pratensis Control 5
Poa pratensis Self 28
Poa pratensis Other 135
Pascopyrum smithii Control 5
Pascopyrum smithii Self 31
Pascopyrum smithii Other 135
Panicum virgatum Control 5
Panicum virgatum Self 30
Panicum virgatum Other 135
Sorghastrum nutans Control 5
Sorghastrum nutans Self 30
Sorghastrum nutans Other 135
Solidago rigida Control 5
Solidago rigida Self 27
Solidago rigida Other 75
Schizachyrium scoparium Control 5
Schizachyrium scoparium Self 30
Schizachyrium scoparium Other 135

Diversity-Productivity experiment was a replicate of the historic E120 experiment at Cedar Creek. 63 plant communities containing 1 to 16 plant species from the above list were planted in 232 plots.

Sampling strategy

Plant-soil feedback experiment
Plant aboveground biomass was clipped, dried and weighed in October 2018..

Diversity-productivity experiment
In August 2018, plant cover in each plot was assessed by visual estimation, then randomly-selected 15 cm by 150 cm strips were clipped, sorted to species, dried to constant weight at 60 °C and weighed to the nearest 0.1 g. The remaining biomass was then

	clipped, dried and weighed. Composition from the clip strip was then used to estimate composition in the entire plot.
Data collection	Plant biomass was clipped, dried, and weighed by a team led by Leslie Forero. Team members included Logan Korte, Megan Koenig, Paige Gueverra, and Cooper Johnson.
Timing and spatial scale	Plant-soil feedback and diversity-productivity experiments were established in Spring of 2015 and sampled in Fall of 2018.
Data exclusions	No data excluded.
Reproducibility	Our diversity-productivity experiment was a replicate of the historic E120 experiment at Cedar Creek.
Randomization	Plant and or community placement within the plant-soil feedback and diversity-productivity experiment was randomly assigned.
Blinding	Not relevant

Did the study involve field work? ☒ Yes ☐ No

Field work, collection and transport

Field conditions	Soils are sandy and of the Nymore series: mixed, frigid, Typic Udipsamment. During the four years of the study, mean annual precipitation and temperature were 723.0 mm and 6.5° C, which is consistent with the 1963 to 2019 records at the site (769.3 mm and 6.6° C, respectively).
Location	45.403290 N, 93.187411 W
Access & import/export	Work performed at the Cedar Creek Ecosystem Science Reserve was done under permit and with full permission and consent of the reserve.
Disturbance	Once the experiment was finished, we removed root barrier using a tractor and seeded the experimental area with native grasses and forbs to prevent weedy encroachment.

Reporting for specific materials, systems and methods

We require information from authors about some types of materials, experimental systems and methods used in many studies. Here, indicate whether each material, system or method listed is relevant to your study. If you are not sure if a list item applies to your research, read the appropriate section before selecting a response.

Materials & experimental systems

n/a	Involved in the study
☒	☐ Antibodies
☒	☐ Eukaryotic cell lines
☒	☐ Palaeontology and archaeology
☒	☐ Animals and other organisms
☒	☐ Human research participants
☒	☐ Clinical data
☒	☐ Dual use research of concern

Methods

n/a	Involved in the study
☒	☐ ChIP-seq
☒	☐ Flow cytometry
☒	☐ MRI-based neuroimaging
